# Soil microbiota and microarthropod communities in oil contaminated sites in the European Subarctic

**DOI:** 10.1038/s41598-021-98680-8

**Published:** 2021-10-04

**Authors:** E. N. Melekhina, E. S. Belykh, M. Yu. Markarova, A. A. Taskaeva, E. E. Rasova, O. A. Baturina, M. R. Kabilov, I. O. Velegzhaninov

**Affiliations:** 1 Institute of Biology, Komi Scientific Center, Ural Branch of Russian Academy of Sciences (IB FRC Komi SC UB RAS), Kommunisticheskaya 28, 167982 Syktyvkar, Russia; 2 Institute of Chemical Biology and Fundamental Medicine, Siberian Branch of the Russian Academy of Sciences (ICBFM SB RAS), Lavrentieva 8, 630090 Novosibirsk, Russia

**Keywords:** Microbiology, Zoology, Ecology, Environmental sciences

## Abstract

The present comprehensive study aimed to estimate the aftermath of oil contamination and the efficacy of removing the upper level of polluted soil under the conditions of the extreme northern taiga of northeastern European Russia. Soil samples from three sites were studied. Two sites were contaminated with the contents of a nearby sludge collector five years prior to sampling. The highly contaminated upper soil level was removed from one of them. The other was left for self-restoration. A chemical analysis of the soils was conducted, and changes in the composition of the soil zoocoenosis and bacterial and fungal microbiota were investigated. At both contaminated sites, a decrease in the abundance and taxonomic diversity of indicator groups of soil fauna, oribatid mites and collembolans compared to the background site were found. The pioneer eurytopic species *Oppiella nova*, *Proisotoma minima* and *Xenyllodes armatus* formed the basis of the microarthropod populations in the contaminated soil. A complete change in the composition of dominant taxonomic units was observed in the microbiota, both the bacterial and fungal communities. There was an increase in the proportion of representatives of Proteobacteria and Actinobacteria in polluted soils compared to the background community. Hydrocarbon-degrading bacteria—*Alcanivorax*, *Rhodanobacter ginsengisoli*, *Acidobacterium capsulatum*, and *Acidocella*—and fungi—*Amorphotheca resinae* abundances greatly increased in oil-contaminated soil. Moreover, among both bacteria and fungi, a sharp increase in the abundance of uncultivated organisms that deserve additional attention as potential oil degraders or organisms with a high resistance to oil contamination were observed. The removal of the upper soil level was partly effective in terms of decreasing the oil product concentration (from approximately 21 to 2.6 g/kg of soil) and preventing a decrease in taxonomic richness but did not prevent alterations in the composition of the microbiota or zoocoenosis.

## Introduction

The use of oil as one of the main sources of energy in the modern world has led to the contamination of territories with oil products as a result of "black gold" spills. The problem of recultivation of such territories is especially acute in countries with a cold climate due to the vulnerability of northern ecosystems and their slow recovery. Changes in oil properties at low temperatures—an increase in viscosity, a decrease in the rate of evaporation of volatile components and a corresponding decrease in the rate of biodegradation—should also be taken into account^1^.

Oil hydrocarbons enter the soil, change its physical and chemical properties, reduce fertility and significantly suppress the activity of soil biota^2–5^. Soil particles become hydrophobic; as a result, they do not let water and air enter the soil, thereby disrupting the habitat conditions of the soil microorganisms, invertebrates, and plants. Complete degradation of individual genetic soil horizons can be observed, and the most vulnerable horizon is the humus horizon^2,6^.

Microbiota perform various functions in the soil that are related to maintaining its homeostasis, including the transformation of organic substrates and mineral elements^7^. In oil-contaminated soil, microorganisms are involved in the processes of utilization of oil and oil products^1,8^.

However, in uncontaminated environments, the share of representatives of microbial communities that are capable of transforming hydrocarbons can comprise only 0.1% whereas their abundance can be significant in oil-contaminated substrates^1^. Decontamination of the soils from oil and oil products is carried out using remediation measures^3,9,10^, often including microbiological preparations based on oil-oxidizing microorganisms (both bacteria and fungi), the use of which is effective in extreme environmental conditions^1,11–13^.

Recently, a number of studies have been carried out on changes in the composition of the microbiome of oil-contaminated soils based on 16S rRNA amplicon sequencing^14–16^. However, although both prokaryotic organisms and some species of fungi are known to be involved in the processes of oil biodegradation^12,17,18^, there have been very few studies of changes in the fungal community.

Multiyear studies of contamination, self-restoration and recultivation of the territories, as well as determination of the efficacy of various methods of ecosystem remediation, are being carried out^3,17–19^ in the conditions of the extreme northern taiga of the European part of Russia. The long-term monitoring of biotic and abiotic components of ecosystems is informative to assess the state of oil-contaminated soils and determine the efficacy of remediation measures in specific conditions^18,19^. A number of indicators, including the abundance of the main trophic groups of microorganisms (oil-oxidizing, ammonifying, nitrifying, oligonitrophilic), the activity of enzymes (catalase, dehydrogenase, urease, cellulase) in the soil, phytocenosis composition and structure^17–19^ and the abundance and structure of groups of soil invertebrates^20,21^ are suggested diagnostic parameters.

The advent of metagenomics^22^ and the revolutionary growth of sequencing efficiency in the second decade of this century have opened up new opportunities for better characterization and comparison of microbiomes of various types of intact and damaged soils^23^. Studies of the microbiomes of soils contaminated with oil or oil products are extremely important. First, such studies allow for us to assess the effects of contamination on the fundamental functional relationships that form the soil ecosystem^24^; second, they allow for us to determine the efficacy of different remediation measures for different soil types and climatic conditions^25,26^. Third, such studies are one of the best ways to search for microorganisms and communities that are capable of degrading hydrocarbons, which can be of high practical value^15^.

Metataxonomics was used here for the first time in combination with soil zoology and analytical chemistry methods to assess the consequences of oil contamination for subarctic soils and determine the efficacy of their remediation. Our aim was to characterize the chemical and biological changes in the soil 5 years after its contamination with oil products under self-restoration conditions and after removing the upper soil layer, as well as assess changes in the microbiome and soil microfauna structure and taxonomic composition.

## Materials and methods

### Study area

The study was carried out in the subarctic part of European Russia—Usinsk Urban Okrug, Komi Republic (66° 11′ 07′′ N, 57° 22′ 08′′ E—control site; 66° 11′ 01′′ N, 57° 22′ 14′′ E—contaminated sites). The study area is located in the forest–tundra and far northern taiga subzones^27^. The climate type is moderately continental. The average annual temperature is 4 °C; January temperatures range from –18 to –20 °C; and the average July temperature is + 14 °C. The snow blanket covers the ground 210 Days a year: from 20 October to early June^28^. Most of the study area belongs to the Pechora–Usinsk region of swamp–podzolic, gley–podzolic, tundra–swamp, and swamp peat soils^29^.

### Study site descriptions

The vegetation cover of the background area is a birch-spruce shrub-green moss forest. The tree layer is dominated by *Picea obovata* (8–10 m high) and *Betula pubescens* (5–7 m high). The undergrowth consists of birch and spruce up to 2–3 m high. The underwood is represented by *Juniperus communis* up to 1 m high, *Sorbus aucuparia* 3–4 m high, *Salix caprea* 2–3 m high, and *Salix phylicifolia* and *Betula nana* 1 m high. The total projective cover of the herb-and-dwarf shrub layer is 60–70%.

Among the dwarf shrubs are *Empetrum hermaphroditum, Vaccinium vitis—idaea, Vaccinium myrtillus, Vaccinium uliginosum, and Ledum palustre*. Herbaceous plants comprise the following species: *Avenella flexuosa, Carex globularis, Luzula pilosa, Melampyrum pratense, Chamaenerion angustifolium, Hieracium altipes, Poa pratensis, Omalotheca sylvatica, and Tussilago farfara*. The moss-lichen cover (with total projective cover of 60–70%) is represented by *Pleurozium schreberi* (50–55%) and *Polytrichum commune* (5–10%) mosses and *Cladina stellaris* lichen. The soil of the site is peaty (Histosol)^30^, and the soil moisture is up to 85%.

The territory represents a sludge collector area with a landfill where equipment for cleaning oil-contaminated soils and grounds is located, as well as a non-hydro-insulated collector of oil sludge. Spring floods accompanied by heavy rains in 2013 resulted in damage to the embankment of the landfill and contamination of the adjacent area with oil products. The soil was contaminated to a depth of 45 cm.

The removal of the upper soil layer contaminated with oil was carried out at the contaminated site, and mineral fertilizers (Azofoska (Fertica, Russia) with NPK 16:16:16 and superphosphate (Fertica, Russia) with Ca(H_2_PO_4_)_2_·H_2_O at 350 and 150 kg/ha, respectively) were added. Sowing of recultivating herbs was not carried out. Biopreparations were not used. Part of the contaminated area was left for self-restoration^18^ and no work was carried out there.

### Soil sample collection

Soil sampling for the examination of the zone affected by the sludge collector site was carried out in September 2018 at three sites: 1—Self-restoration (SR), 2—Removal of the upper soil layer (R), and 3—an Undisturbed Forest (UF) site of a birch-spruce forest located in the immediate vicinity of the SR and R sites in similar edaphotopic conditions that was not subjected to oil contamination or other anthropogenic influences.

The SR and R sites completely lost vegetation cover after the oil spill in 2013. The projective cover on the territory contaminated with the contents of the sludge collector was 10–15 and 30–40% at the SR and R sites, respectively, during the time of sampling. At the SR site, two variants of the soil were examined: with and without vegetation cover. Sampling was conducted 5 years after contamination.

**Figure 1:**
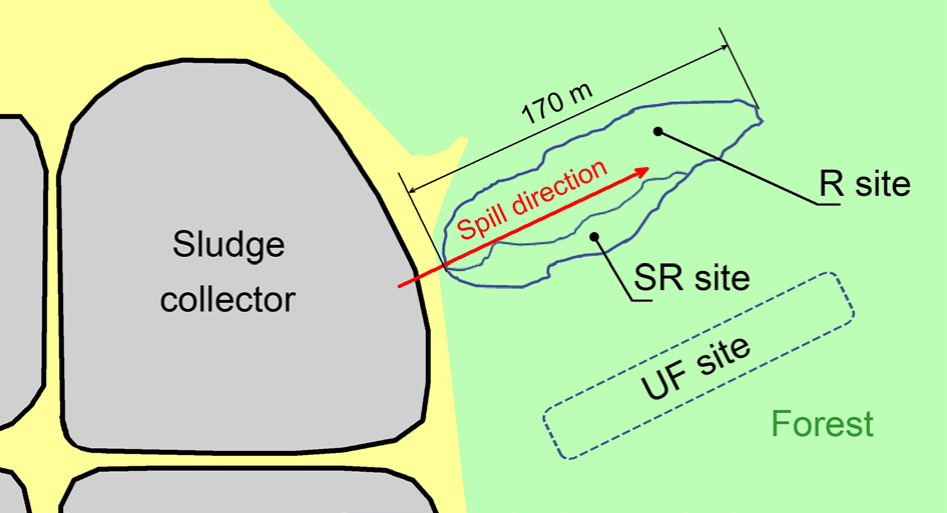
Scheme of the oil spill and location of experimental sites. SR—Self-restoration, R—removal of the upper soil layer, and UF—undisturbed forest. The scheme is based on an image taken with a quadcopter.

Soil samples for chemical analysis were collected at the same sites from which samples were collected for the metataxonomic analysis and soil fauna composition determination. Each sample plot was 0.2 × 0.2 m and located approximately 10 m from each other along the oil spill. The site layout is shown in Fig. 1. The soil columns (7 cm deep) were collected from all four corners and the centre of the plot before pooling and mixing the samples well. Part of the mixed soil was used as a sample for metataxonomic analysis, and another part was used for chemical analysis. A total of 46 samples were collected (16 samples at the UF site and 15 samples each from SR and R). All were used for metataxonomics analysis. Nine per study site (27 in total) were used for chemical analysis. Sampling for analysis of invertebrate fauna was performed in adjacent plots. Twelve 5 × 5 cm by 10 cm deep soil samples^31^ were collected at each site.

### Soil chemical analyses

Chemical analysis of the soils was carried out in the “Ecoanalit” laboratory of IB FRC Komi SC UB RAS. The following characteristics of the soils were determined: pH of the aqueous extract^32^, total soil carbon and nitrogen content using gas chromatography (CHNS-O, CE Instruments, Italy. FR.1.31.2016.23502), and total petroleum hydrocarbons (TRN) using gravimetry (RD 52.18.647–2003).

### Soil invertebrates

The collected soil samples were immediately delivered to the IB FRC Komi SC UB RAS and placed into Tullgren soil extractors^33^. The microarthropod fauna was extracted under 40 Wight bulbs in 96% alcohol for seven to ten days until the soil was completely dry.

Collembola species were identified according to their morphological taxonomic characteristics^34–37^. Oribatid mites were identified as in ref.^38^. The taxonomy of oribatid mites is given according to the classification of Subías^39^.

The identified springtail species were also assigned to one of three functional groups with respect to their vertical distribution (epiedaphic, hemiedaphic, and euedaphic) and according to their trophic guilds (epigeic plant and microorganism consumers, EPMC; epigeic animal and microorganism consumers, EAMC; hemiedaphic microorganism consumers, HMC; and euedaphic microorganism consumers, EMC)^40^. The oribatid mite species were assigned to one of six life forms: epiedaphic, hemiedaphic, euedaphic, eurybiontic, hydrobiontic, and nonspecialized species.

The significance of differences (with p < 0.05) in the abundance and α-biodiversity indices of microarthropods was determined using the Mann–Whitney U-test in PAST V 3.0 software.

### Soil metataxonomics analysis

To isolate the total DNA, the samples were preliminarily centrifuged (2,500 g, 10 min), alcohol was removed, and the pellets were dried. The total DNA was extracted using the DNeasy PowerSoil Kit (Quagen, USA) according to the manufacturer’s instructions. Bead beating was performed using TissueLyser II (Qiagen) for 10 min at 30 Hz. The quality of the DNA was assessed using agarose gel electrophoresis.

The 16S rRNA gene and ITS2 regions were amplified with the primer pairs V3/V4 and ITS3_KYO2/ITS4, respectively, combined with Illumina adapter sequences (Fadrosh et al., 2014). PCR amplification was performed as described previously^41^. A total of 200 ng PCR product from each sample was pooled and purified using a MinElute Gel Extraction Kit (Qiagen, Germany). The obtained amplicon libraries were sequenced with 2 × 300 bp paired-ends reagents using MiSeq (Illumina, USA).

Raw sequences were analysed with the UPARSE pipeline^42^ using Usearch v11.0.667. The UPARSE pipeline included merging of paired reads, read quality filtering, length trimming, merging of identical reads (dereplication), discarding singleton reads, removing chimaeras and OTU clustering using the UPARSE-OTU algorithm^43^. The OTU sequences were assigned a taxonomy using the SINTAX^44^ and 16S RDP training set v16^45^ and fungi ITS UNITE v.8.2^46^ as references. As a result of the analysis performed, 730,469 reads of the 16S rRNA gene fragment and 1,315,679 reads of the ITS were obtained.

Alpha diversity metrics were calculated in Usearch. Rarefaction and extrapolated curves were generated using the “iNEXT” package^47^. The Mann–Whitney test was performed using the Python scientific computing library SciPy (v.1.5.1).

### Statistical analyses

**Figure 2:**
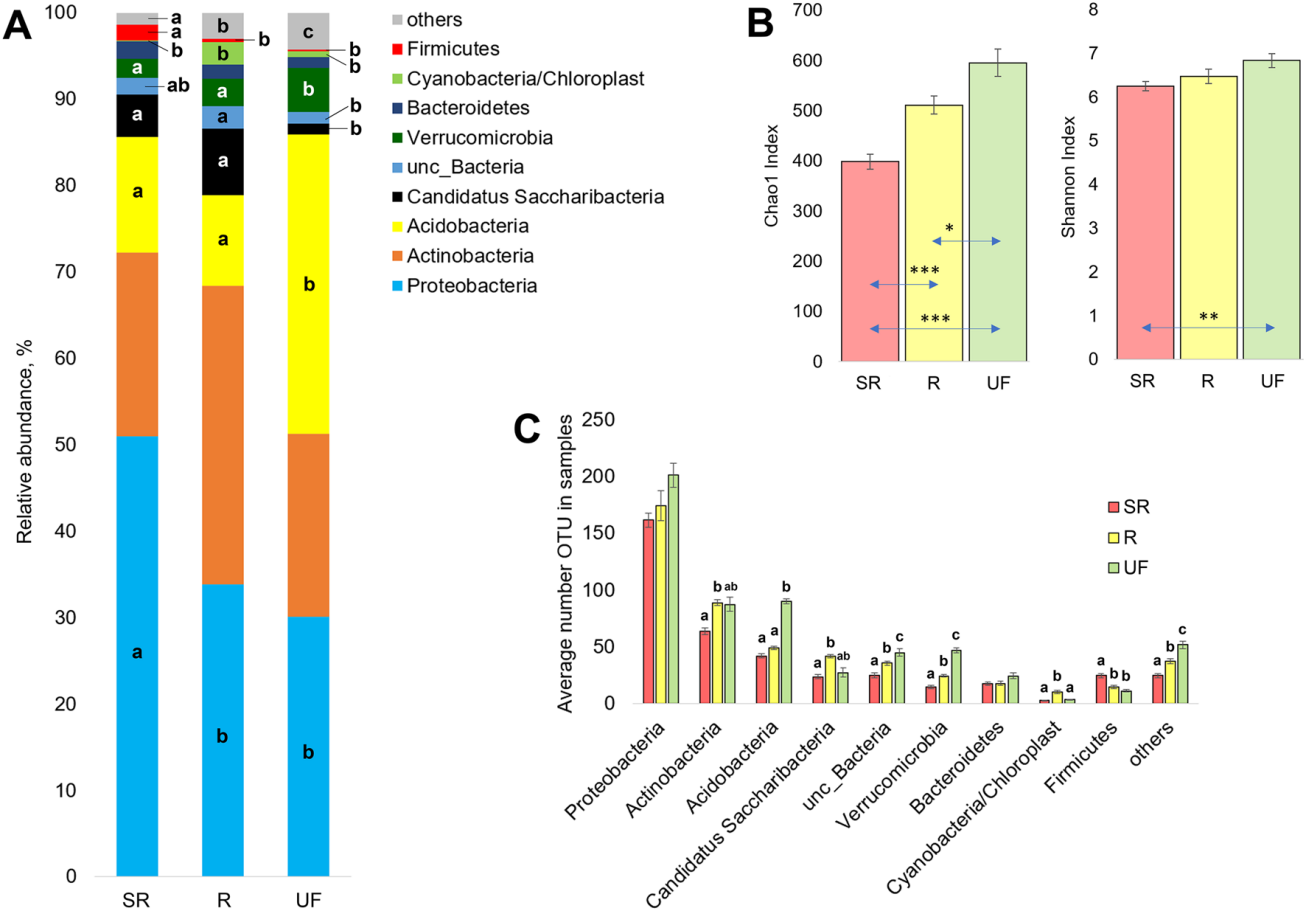
Taxonomic structure and richness of the soil microbiome of the study areas based on total 16S rRNA sequencing. (**A**) Averaged values of the phylum distribution of annotated reads in samples from the experimental sites. (**B**) Averaged values of the Chao1 and Shannon indices in samples of the study areas. (**C**) Average number of OTUs found in samples of experimental sites within the corresponding phyla. Lowercase letters indicate significant differences between the corresponding indicators of different experimental sites at p < 0.05 (Mann–Whitney test with FDR correction). In diagram (**B**), *, ** and *** denote significant differences at p < 0.05, 0.01, and 0.001, respectively (Mann–Whitney test).

**Figure 3:**
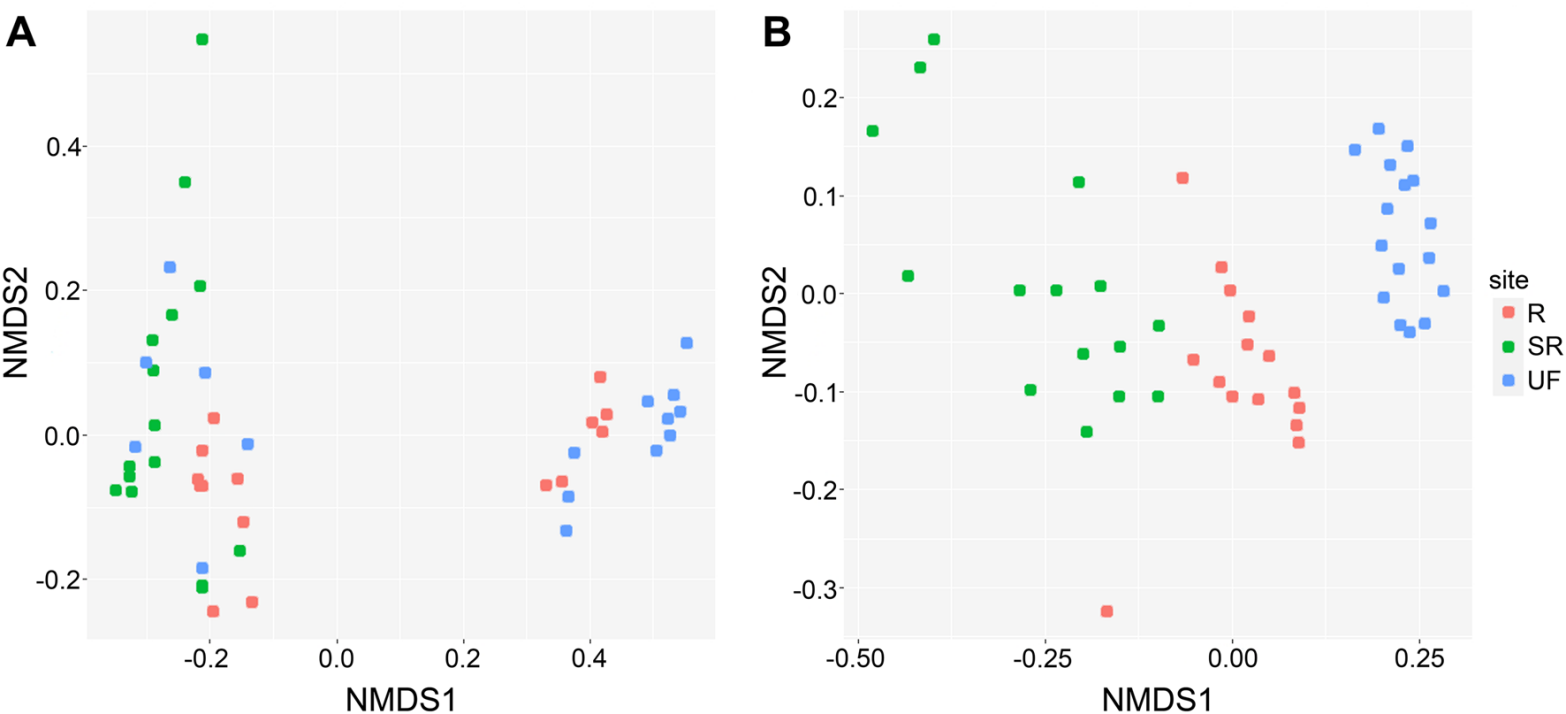
Nonmetric multidimensional scaling (NMDS) ordination plots based on the composition of bacterial (**A**) and fungal (**B**) taxa with Bray–Curtis distance among samples.

**Figure 4:**
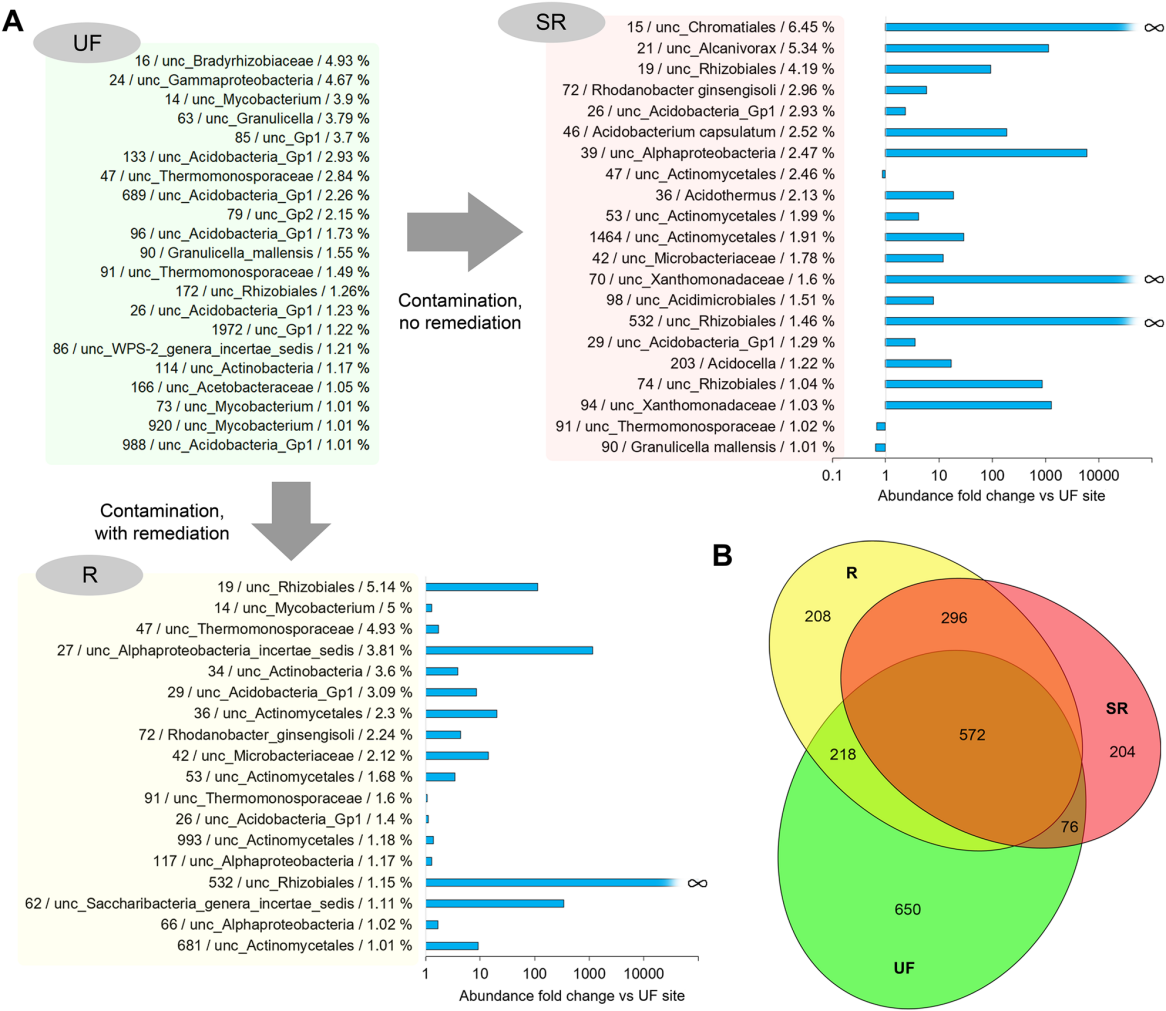
The most represented OTUs (more than 1% of reads) at the study sites based on sequencing of 16S rRNA amplicons and differences in the number of reads at the SR and R sites relative to the UF site. The number before the “/” indicates the OTU number within the study (and in Supplementary file 1). The average percentage of reads corresponding to the indicated OTU in the samples of the site is shown after the OTU classification. The diagrams (logarithmic scale) show the fold change of the indicator relative to the average percentage of reads of the OTU in the samples from the UF site. The $\propto$ symbol indicates that the fold change can be considered equal to infinity since no reads corresponding to this OTU were detected in the samples from the UF site. (**B**) The number of common and unique OTUs detected at the experimental sites. All OTUs were taken into account, including those represented by one read in one of the samples from the corresponding site.

**Figure 5:**
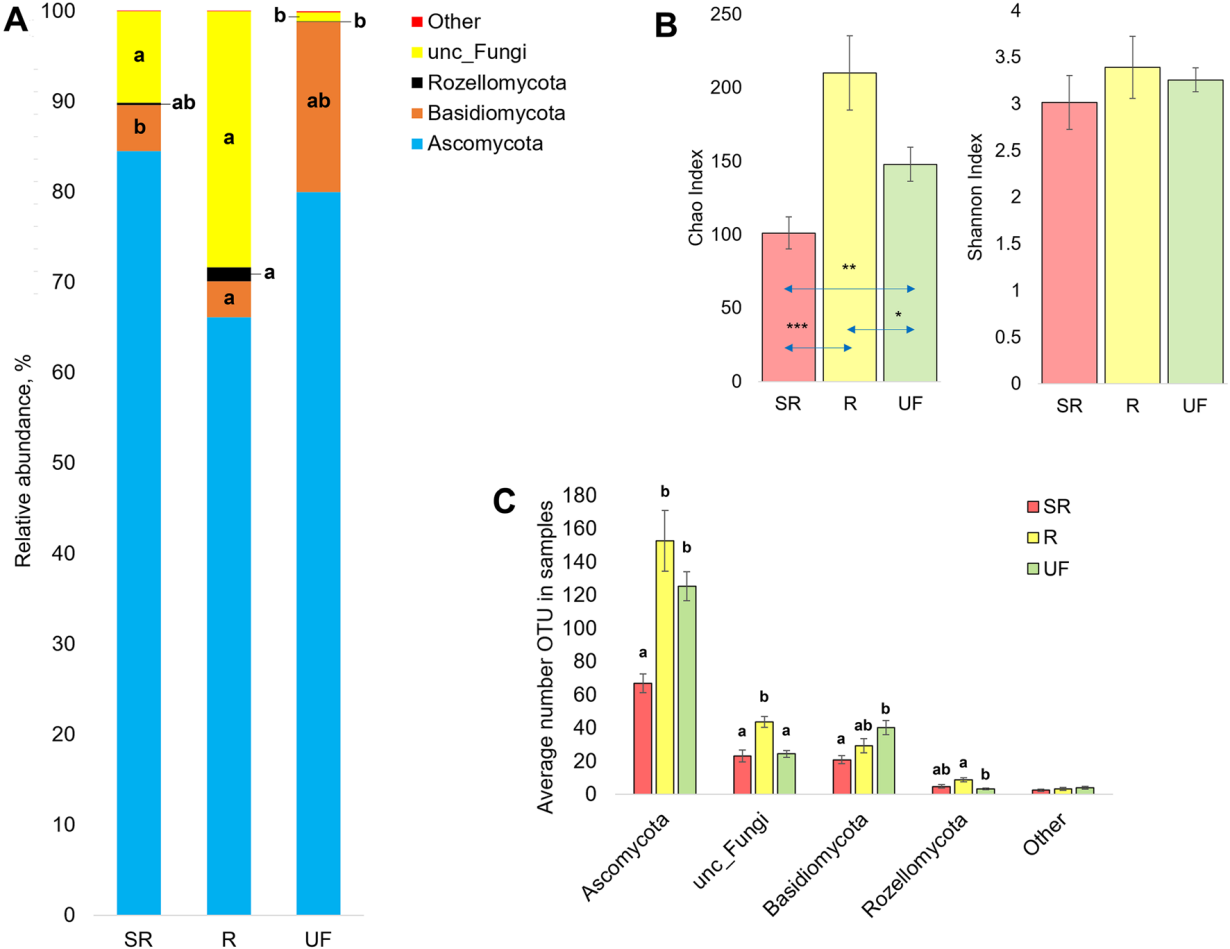
Taxonomic structure and richness of the soil microbiome of the study areas based on total ITS sequencing. (**A**) Averaged values of the phylum distribution of annotated reads in samples from experimental sites. (**B**) The averaged values of the Chao1 and Shannon indices in samples of the study areas. (**C**) The average number of OTUs found in the samples of the experimental sites within the corresponding phyla. Lowercase letters indicate significant differences between the corresponding indicators of different experimental sites at p < 0.05 (Mann–Whitney test with FDR correction). In diagram (**B**), *, ** and *** denote significant differences at p < 0.05, 0.01, and 0.001, respectively (Mann–Whitney test).

**Figure 6:**
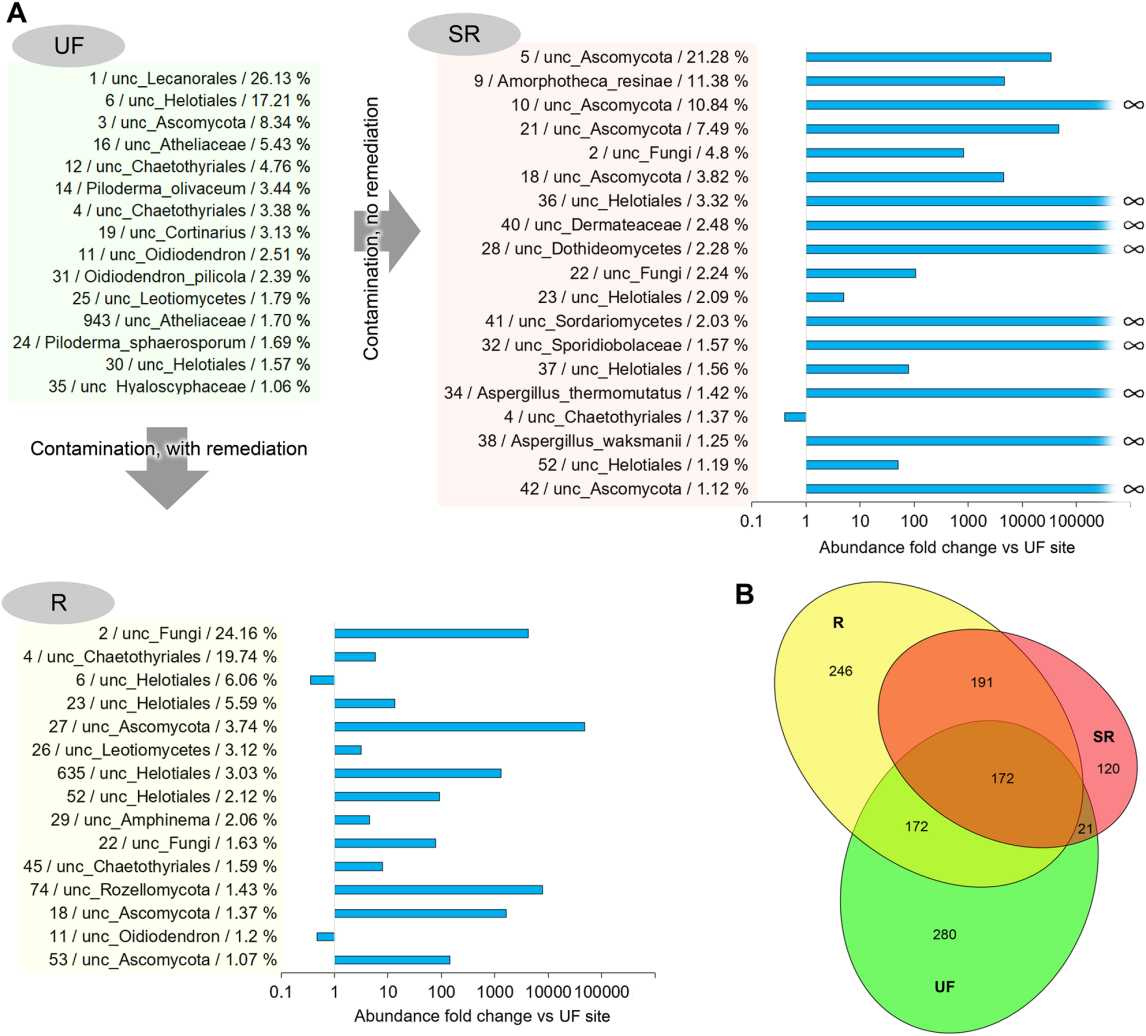
(**A**) The most represented OTUs (more than 1% of reads) at the study sites based on sequencing of ITS amplicons and differences in the numbers of reads at SR and R sites relative to the UF site. The number before the “/” indicates the OTU number within the study (and in Supplementary file 2). The average percentage of reads corresponding to the indicated OTU in samples of the site is shown after OTU classification. The diagrams (logarithmic scale) show the fold change of the indicator relative to the average percentage of reads of the OTU in the samples from the UF site. The $\propto$ symbol indicates that the fold change can be considered equal to infinity since no reads corresponding to this OTU were detected in the UF site samples. (**B**) The number of common and unique OTUs detected at the experimental sites. All OTUs were taken into account, including those represented by one read in one of the samples from the corresponding site.

Statistical processing of the data was carried out using Microsoft Office Excel and the Statistica 6.0 software package. The tables show mean values and standard deviations. The values of the Chao-1 richness estimator and Shannon biodiversity index calculated for each individual sample were averaged to obtain the value characterizing the site under study. All values of the fractions of reads annotated for an OTU were also averaged among the samples of a site. Differences in biodiversity indices of experimental sites and differential abundances were assessed using the Mann–Whitney test. All multiple comparisons were made using false discovery rate (FDR) correction. OTUs that accounted for more than 1% of all 16S rRNA or ITS reads were considered dominant. A nonmetric multidimensional scaling (NMDS) analysis was performed with the R program^48^ to illustrate the sampling site similarity based on a Bray–Curtis dissimilarity assessment in terms of the bacterial or fungal species composition using the metaMDS function^49^. Data charts were built using R program (Fig. 2) and Microsoft Office Excel (Figs. 3, 4, 5, 6). Rastering, layout and design of all figures was carried out using the Photoshop software (Adobe, USA).

## Results and discussion

### Soil chemical properties

The total soil carbon and nitrogen content, pH and total petroleum hydrocarbons (TPH) in the soils of the study sites are presented in Table 1. The acidity of the soil at the UF site varied from 4.4 to 5.1, the nitrogen content varied from 0.65 to 1.45% and the carbon content varied from 20 to 45%, which is typical for soils of the taiga zone^31^. The acidity of the soils in sites contaminated with TPH was generally slightly higher and varied from 4.6 to 5.6 (Table 1). The nitrogen and carbon content were significantly (p < 0.005) lower than those at the UF site. When soil is severely contaminated with oil, its pH is known to increase due to a weakly alkaline or neutral reaction of oils^50^. The content of biogenic elements may decrease because sites SR and R were deprived of vegetation as a result of significant contamination with oil products.

**Table 1:** Total soil carbon and nitrogen content, pH and TPH in the contaminated (SR, R) and uncontaminated (UF) study sites (mean ± SD).

Site	pH	N, %	C, %	TPH, mg/kg
SR	5.1 ± 0.2	0.6 ± 0.3	18.5 ± 7.4	21,450 ± 11,033
R	5.0 ± 0.4	0.3 ± 0.2	10.7 ± 5.3	2608 ± 1251
UF	4.6 ± 0.2	1.1 ± 0.3	34.8 ± 9.3	1002 ± 837

In general, the content of oil products in the surface layer of the soil of the UF site (Table 1) is enhanced compared with the background content of hydrocarbons (11–32 mg/kg) in the soils of the studied region^50^, although it does not typically exceed the permissible level. The value of this indicator at different sampling points varied from 270 to 2,900 mg/kg; the level of contamination was assessed as low in two samples and medium in one^51^. The increased content of oil products in the soils of the undisturbed site is associated with the lateral runoff of the contents of the sludge collectors from the adjacent contaminated landscapes, which is typical for bog-podzolic soils occupying accumulative landscapes^50^.

The level of soil contamination with oil products at the recultivation site at the time of sampling was assessed as average^51^; however, in several samples, the hydrocarbon content exceeded 3,000 mg/kg. Contamination of the territory of the self-restoration site (Table 1) was characterized as very high^51^.

### Soil microarthropods

#### Oribatid mites

Soil invertebrates react to anthropogenic influences on natural ecosystems, causing changes in the composition, structure and abundance of populations^52–56^. This determines their value as bioindicators^57–59^.

While conducting multiyear studies, certain patterns in the dynamics of soil invertebrates in oil-contaminated ecosystems^20,21^ were identified. The main taxa of microarthropods were found to follow different trends in changes in their abundance: a decrease for *Diptera* larvae and mesostigmatid mites and an increase for springtails and oribatid mites. The stages of recovery of soil zoocoenosis were established; the taxonomic groups of soil microarthropods that serve as biomarkers of stages of succession were identified: for the first stage, *Diptera* larvae and mesostigmatid mites; for the second stage, collembolans; and for the third stage, oribatid mites^21^. The relationship between the dynamics of the abundance, composition and structure of groups of soil invertebrates and the succession of the plant community under conditions of oil contamination^60^ were established. The dependence of the succession of the zoocoenosis on the methods of remediation was identified; the most successful recovery of soil zoocoenosis in the soil occurred when biopreparations were used^19,21^.

Thirty species of oribatid mites from 24 families were found in the territory studied, including 29 species from 24 families at the undisturbed site (Table S1). The whole spectrum of life forms of oribatid mites typical for taiga forests was represented. The epiedaphic life form (inhabitants of the soil surface and upper horizons of the litter, according to the D.A. Krivolutsky’s classification of life forms^33^) was distinguished by the greatest diversity of species. Among the epiedaphic species, the most abundant were *Ceratozetes gracilis, Nanhermannia sellnicki*, *Eueremaeus oblongus silvestris, Eupelops plicatus* and *Chamobates pusillus*. Some samples had a noticeable presence of hemiedaphic species (inhabitants of the litter, according to D.A. Krivolutsky): *Heminothrus longisetosus* and *Camisia biurus*.

Additionally, euedaphic species were observed, the inhabitants of small soil wells (according to D.A. Krivolutsky^33^), and *Oppiella nova* and *Oppiella neerlandica* were dominant (Table S1)*.* The most numerous of the eurybiontic species was *Tectocephes velatus*. In the background community, nonspecialized (*Palaeacarus hystricinus, Hypochthonius rufulus, Liochthonius (L.) sellnicki*) and hydrobiontic (*Malaconothrus (M.) monodactylus*) species (according to D.A. Krivolutsky^33^) were also observed, but the hydrobiontic species had low abundance.

Such a spectrum of life forms of oribatids is characteristic of an undisturbed community. The inhabitants of the soil surface were repeatedly shown to be the most vulnerable to various types of soil disturbance^57,61^. The abundance of preimaginal stages of oribatid mites, i.e., larvae and nymphs, was high in undisturbed soil of the background community (31,100 ± 5655 ind./m^2^).

Oribatid mites were rare at the sites with oil contamination, both R and SR. Species of the family *Oppiidae* were found there; most often, the species *Oppiella nova*. *O. nova* is known as eurytopic, tolerant to different community disturbances and registered as a pioneer species in recovery successions^21,54^. In addition to *O. nova,* sporadic representatives of the eurybiontic species *Scheloribates laevigatus*, *Zygoribatula exilis* and *Tectocepheus velatus* and the epiedaphic species *Epidamaeus bituberculatus* were also found at the recultivation site (Table S1). The abundance of the preimaginal stages of oribatid mites at the recultivation site was 267 ± 150 ind./m^2^. The self-restoration site had the lowest number of species compared with the background and recultivation sites. In addition to *O. nova* mentioned above, *S. laevigatus* and *Oribatula tibialis* were also found there*.*

The very low abundance of oribatid mites at the contaminated sites indicates a significant soil disturbance. E.N. Melekhina^21^ established that oribatid mites are indicators of a later (third) stage of microarthropod recovery succession. Numerous studies have shown that after disturbances of various types, Oribatida restore their diversity and abundance more slowly than other mass groups of invertebrates^55,56^. In particular, they were very sensitive to oil contamination of soil^62,63^. It can be concluded that, five years after remediation, the oribatid mite communities were at the earliest stages of recovery succession.

#### Collembola

In total, 2238 individuals of Collembola belonging to 24 species were found and identified. A complete list of the identified species and their abundance is provided in Table S2. The mean total abundance of springtails significantly differed across all sites. The background site (UF) was characterized by the highest total abundance of collembolans in comparison with the R and SR sites. The lowest total abundance was found at the contaminated site where recultivation was carried out. The total abundance of collembolans was much higher at the self-restoration site than in the recultivated soil (Table S2).

The species richness of springtails also significantly differed between the study sites (Table S2). The lowest number of species was recorded in the sites with oil contamination, both the recultivation and self-restoration sites (Table S2). It was the highest at the UF site. The mean species richness at sites R and SR was significantly lower than that at UF.

In the spectrum of life forms of the background site, a sharp predominance of the hemiedaphic life form (more than 80% of all Collembola) was observed, which is characteristic of undisturbed soils; one species, *Folsomia quadrioculata*, was dominant.

At the R site, only two groups were notable in the spectrum of life forms: the hemiedaphic group, which was predominant in abundance, and the epiedaphic group. The dominant species included two species, namely, *Proisotoma minima* and *Xenyllodes armatus*.

The abundance of epiedaphic springtails significantly differed at sites SR and UF. Hemiedaphic collembolans were significantly affected by “Site type”. The abundance of this group at the site with recultivation was decreased compared with that at the self-restoration and control sites. Euedaphic springtails were found only in uncontaminated soil (Table S2).

Among trophic groups of collembolans, “Site type” had a significant effect on epigeic animal and microorganism consumers (EAMC), hemiedaphic microorganism consumers (HMC) and euedaphic microorganism consumers (EMC) (Table S2).

Our results demonstrate that the recovery of the total abundance of springtails and the abundance of selected functional groups at the SR and R sites was very slow. Similar but stronger effects of recultivation methods after oil contamination were also reported in forest-tundra ecosystems^17^. However, the magnitude of differences was much larger than in our case.

The significant reduction in the number of species at the self-restoration and remediation sites in comparison with the control plot indicates that it takes longer to restore the species richness of springtails than their total abundance. This suggests that a limited number of relatively resistant and ecologically flexible species of collembolans, which can colonize disturbed soils contaminated by oil, mainly contribute to the restoration of the total population at the self-restoration site. A similar picture was shown for rice-growing systems^62^.

At the self-restoration site, *Proisotoma minima* was found to be absolutely prevalent, accounting for more than 90% of all the species specimens. This species is common at the initial stages of vegetation restoration and is tolerant of a high level of contamination^36^. Similar patterns have been shown for ecosystems subject to other disturbance types^62,63^. The lack of significant differences in the springtail species richness between the SR and R sites is also interesting. In our opinion, this may be due to the development of vegetation cover, which causes changes in bacterial and fungal communities^64^ that are the main source of food for springtails^40^. In addition, mechanical removal of the upper soil horizon leads to soil compaction, which affects the living space of euedaphic collembolans^65^.

Our studies show that there are no significant differences in the abundance of epiedaphic springtails between the recultivation and control plots. Notably, a significantly low density of epiedaphic collembolans found in the self-restoration site indicates extreme conditions. The data obtained can be confirmed by the epigeic trophic guilds that consume animals and microorganisms, since their abundances differed at the self-restoration and control sites. This indicates that this group is adapted to the rapid environmental changes in tilled soils and thus can quickly recolonize soil after the end of intense disturbance, which is in line with the data obtained for rice paddies^62^. A negative effect of the site type was found for the abundance of euedaphic springtails, including relevant trophic guilds. Our data confirm the assumption that representatives of the euedaphic life form have a selective advantage in disturbed soils due to the development of complex sensory organs^66^ as well as their habitat dependence on the microstructure of soil pores and level of soil compaction^65^.

#### Other groups of arthropods

In addition to oribatid mites and springtails, the dominant taxonomic groups in the background, i.e., undisturbed community, included mesostigmatid mites (8200 ± 1399 ind./m^2^). *Coleoptera* larvae (1133 ± 108 ind./m^2^) and *Diptera* larvae (1133 ± 157 ind./m^2^), prostigmatid mites (namely, *Trombidiidae*) (1000 ± 285 ind./m^2^), uropod mites (567 ± 289 ind./m^2^) and thrips (667 ± 320 ind./m^2^) were also found.

The zoocoenosis was more diverse at the recultivation site than at the self-restoration site. In addition to oribatid mites and springtails, beetles were found at the R site, both imago and larvae (33 ± 33 ind./m^2^ and 233 ± 91 ind./m^2^, respectively), *Diptera* larvae (467 ± 146 ind./m^2^), *Hymenoptera* larvae (33 ± 33 ind./m^2^), thrips (767 ± 305 ind./m^2^), spiders (167 ± 77 ind./m^2^), *Prostigmata* (267 ± 102 ind./m^2^), *Uropodida* (67 ± 45 ind./m^2^) and *Acaridia* (133 ± 102 ind./m^2^). Mesostigmatid mites were the most abundant (4333 ± 1056 ind./m^2^). At the SR site, Mesostigmata (5100 ± 1138), Prostigmata (133 ± 90), Diptera larvae (1800 ± 741) and Aranea (33 ± 33) were found.

### Soil microbiome

#### Bacteria

The analysis of the obtained results indicates the greatest α-diversity of the bacterial community at the undisturbed site. The Chao1 index was significantly higher there than at both contaminated sites (Fig. 2B). However, the number of bacterial OTUs at the recultivation site was reduced to a lesser extent than at the self-restoration site. When the diversity was assessed using the Shannon index, statistically significant differences were found only between the SR and UF sites.

It is interesting that under different conditions of contamination (extent, type of contaminant, duration of contamination, type of soil and climatic conditions), the species diversity of bacteria in oil-contaminated areas can either decrease^67–70^ or increase^71–73^; the changes can be very pronounced in both directions. However, it is worth noting that a decrease in richness in response to oil contamination was observed in studies carried out in Poland and in the far north of Canada whereas an increase was observed in China and Thailand. The authors of the study^74^ noted that in samples of soils contaminated with refined oil, the diversity of bacterial communities was higher than that in samples contaminated with crude oil. Thus, at low/nonextreme contamination levels, it would be more useful to assess the diversity changes as well as variations in the community structure. The mechanisms of changes in diversity and their dependency on the contamination level require separate study at different initial conditions.

Analysis of the NMDS plot allowed for us to separate two distinct clusters (Fig. 3A). The first contains all samples from the SR site, two-thirds of the R site and one-third of the UF site. The second cluster joins most points of the UF site and the rest of the R site. To some extent, the clusters reflect soil contamination with oil products. The bacterial community differed in richness and abundance in the soil of the most contaminated locations of the SR site. Variability and close values in the content of crude oil in the soil of UF and R sites create gradient conditions for the formation of the bacterial community. The intermediate position of the R site community was confirmed with data on phylum representatives’ richness and abundance.

The phyla Acidobacteria, Proteobacteria, and Actinobacteria accounted for more than 80% of the bacterial community (Fig. 2A) at the UF site. The same was reported for undisturbed taiga forests in North America^75,76^. Other phyla were observed to a much lesser extent. Representatives of these three phyla of the bacterial domain were also characterized by the greatest number of OTUs. A great richness of the Verrucomicrobia, Saccharibacteria, and Bacteroidetes phyla was also observed, although they were characterized by a low abundance.

The dominance of the Proteobacteria, Acidobacteria, and Actinobacteria phyla persists at oil-contaminated sites, but the order of dominant phyla changes. The abundance of Actinobacteria significantly decreased in soils from both the SR and R sites, and that of Proteobacteria increased with increasing contamination level. Actinobacteria abundance at the R site increased by 1.5 times compared to that at the UF and SR sites.

The richness and representation of the Verrucomicrobia and Bacteroidetes phyla at the contaminated sites (Fig. 2A,C) were lower than at the UF site. In contrast, the proportion of the Saccharibacteria phylum in the bacterial community structure was significantly higher. The OTU diversity of representatives of Saccharibacteria at the R site was 1.5 times higher than that at the UF site whereas at the SR site it did not differ from that at the UF site level, as in the case of Actinobacteria. The relative abundance of Saccharibacteria at the R site was 5 times higher. The richness and relative abundance of the Firmicutes phylum also increased as the contamination level increased. Interestingly, in some samples collected at the R site, increases in the share (values varied from 0.05 to 16%) and richness (from 2 to 19 OTUs) of Cyanobacteria were observed, which may be associated with a spotty overgrowth of the site with herbaceous plants. Along with areas with a high projective cover of perennial grasses, spots completely devoid of vegetation cover were observed there.

The Proteobacteria phylum is a numerous and heterogeneous group of bacteria, the division of which into classes is based on the analysis of 16S rRNA^77,78^. At all sites investigated, this bacterial phylum had the highest number of detected OTUs. The share of Proteobacteria in the samples increased with increasing contamination levels, despite a decrease in detected OTUs. The increase in Proteobacteria abundance is a common change in the soil microbiome in response to oil contamination, both in soils of southern and temperate latitudes^15,26,67,71,72^ and beyond the Arctic Circle^69,70^. However, there are exceptions to this rule, when the relative abundance of Proteobacteria in a contaminated area becomes significantly lower^73^ or its changes vary ambiguously depending on the extent and history of contamination^68,79^.

Representatives of four classes of this taxon were found in the study area (Table 2): Alpha-, Beta-, Gamma-, and Delta-proteobacteria. More than half of all bacteria of this group at all the sites were Alphaproteobacteria, among which the orders *Rhizobiales* and *Rhodospirillales* could be distinguished; their representatives comprised 30 and 20% Proteobacteria, respectively, at the undisturbed site. The share of *Rhizobiales* in the samples from the most contaminated site was significantly lower (Table 2) whereas the number of certain OTUs at the SR and R sites was the same or slightly higher than that at the UF site.

**Table 2:** Abundance of Proteobacteria OTUs registered at the study sites (mean ± SEM). Different letters indicate a significant difference among data within the row.

Site	Average abundance, %			Average number of OTUs		
	SR	R	UF	SR	R	UF
*Alphaproteobacteria*	52.5 ± 2.5^a^	77.1 ± 3.5^b^	65.6 ± 1.8^b^	92.0 ± 3.3	99.1 ± 6.0	104.9 ± 5.2
Rhizobiales	20.9 ± 1.6^a^	28.8 ± 2.4^ab^	30.1 ± 2.0^b^	22.8 ± 0.7	23.1 ± 1.2	19.9 ± 0.9
Rhodospirillales	9.5 ± 1. 6^a^	12.9 ± 0.9^a^	20.5 ± 1.8^b^	26.2 ± 1.2^a^	29.7 ± 1.2^a^	37.3 ± 1.5^b^
Acetobacteraceae	7.4 ± 1.7^a^	10.3 ± 1.0^a^	17.0 ± 1.6^b^	17.3 ± 0.9^a^	21.3 ± 0.6^b^	24.0 ± 0.8^b^
Rhodospirillaceae	0.5 ± 0.1^a^	0.4 ± 0.1^a^	1.6 ± 0.2^b^	3.2 ± 0.3^ab^	2.7 ± 0.4^a^	4. 6 ± 0.3^b^
*Betaproteobacteria*	3.0 ± 0.6	3.6 ± 0.7	4.5 ± 0.9	13.0 ± 0.9	11.5 ± 1.4	13.9 ± 1.2
*Gammaproteobacteria*	41.8 ± 2.8^a^	17.3 ± 3.2^b^	25.6 ± 1.6^b^	40.9 ± 1.7	49.5 ± 4.3	43.9 ± 2.2
*Deltaproteobacteria*	0.8 ± 0.4^a^	1.5 ± 0.2^b^	3.38 ± 0.74^b^	3.9 ± 0. 6^a^	5.2 ± 0.8^a^	18.3 ± 2.1^b^

A different picture was observed for OTUs that belong to the order *Rhodospirillales*, including the most representative families Acetobacteraceae and Rhodospirillaceae; at the sites contaminated with oil products, both the share of bacteria of these taxa and number of OTUs were lower.

The next most represented class at the study sites was Gammaproteobacteria. Its share was significantly higher at the self-restoration site than in the soil at both other sites. Within the class, the largest increase was observed among the members of the *Xanthomonadales* order. The number of OTUs of Gammaproteobacteria was almost the same at all experimental sites.

Changes in the abundance of Alpha- and Gammaproteobacteria revealed at the contaminated sites were typical for areas contaminated with oil products. However, an increase in Proteobacteria abundance can occur in various ways. For example, only at the expense of Alphaproteobacteria^26^, only at the expense of Gammaproteobacteria^15,69^ or at the expense of all classes of the phylum^68^. In the present study, the richness and share of the total abundance of Betaproteobacteria at the study sites were insignificant. The abundance and OTU number of Deltaproteobacteria were significantly reduced in the contaminated areas.

The Acidobacteria phylum comprised more than 30% of all registered bacteria at the UF site and was second in the number of OTUs. These bacteria are known to be well adapted to living in acidic soils that are poor in organic matter^80^. However, their abundance was three times lower at the oil-contaminated sites, and the OTU diversity was two times lower than at the undisturbed site. This could be due to changes in soil acidity, among other things; the abundance of Acidobacteria within a microbial community was shown to be strongly regulated by pH, significantly decreasing when the pH value exceeds 5.5^80,81^.

Representatives of nine groups of the 26 currently known subdivisions of Acidobacteria^82^ were present in the study area, with the dominant Gp1 that included more than 80% of all Acidobacteria at all sites. The share of this group tended to be higher at the oil-contaminated sites, and the OTU diversity of this group of bacteria was lower by 36 and 43% at the R and SR sites, respectively (Table 3).

**Table 3:** Abundance of OTUs from subdivision Gp1 of the Acidobacteria phylum registered at the study sites (mean ± SEM). Different letters indicate a significant difference among data within the row.

Site	Average abundance, %			Average number of OTUs		
	SR	R	UF	SR	R	UF
Gp1	85.1 ± 2. 7	87.2 ± 1.1	81.1 ± 2.3	30.1 ± 1.0^a^	33.5 ± 1.0^b^	52.8 ± 0.9^c^

Another bacterial phylum that was dominant in the area studied was Actinobacteria. Several representatives of this phylum are known to decompose organic matter in soils^83^. The number of OTUs of this phylum was the lowest at the SR site (Fig. 2C). Actinobacteria representatives of the three classes that comprise this phylum were predominant at all sites studied.

The results presented above are consistent with the results of research on the metagenomes of oil-contaminated soils. Some authors found an increase in the share of Proteobacteria in soil samples, particularly Gammaproteobacteria^14–16,84,85^, which indicates that this is the best-adapted phylogenetic group for hydrocarbon pollution, particularly in cold environments. They also noted an increase in the abundance of Bacteroidetes, Firmicutes, and Actinobacteria; apparently, in acidic soils of cold climatic zones, an increase in the abundance of Actinobacteria was more noticeable whereas in areas with a warm climate and more alkaline soils, the shares of Bacteroidetes and Firmicutes, which were numerous, also increased. The increase in the share of bacteria of the listed groups in oil-contaminated soils is due to the presence of enzymes that allow for their possessors to use oil products as substrates^12,69^.

A bacterial community that effectively uses oil products as a substrate can be built from multilevel metabolic chains, including representatives of different phyla^86,87^. Therefore, from a functional point of view, the changes in the composition of the dominant species and genera of bacteria are most interesting.

The OTUs characterized by reads having more than 1% of the total number analysed in samples from the SR and R sites and the differences relative to the UF site are shown in Fig. 4A. The results allow for us to conclude that the main (in terms of abundance) structure of the bacterial community at the contaminated sites completely changed. It is important to note that the averaged numbers obtained from 15–16 samples from each of the sites are presented, which practically excludes the random nature of the dominance of certain OTUs. An uncultured bacterium of the *Chromatiales* order (Gammaproteobacteria) was represented the most (6.45%) in the contaminated soil of the SR site, to a much lesser extent in the soil that underwent remediation (0.13%) and was not found in the soil of the UF site. Previously, representatives of this order have been found in the soil community characterized by accelerated biodegradation of oil products^88^. However, no detailed studies of the petroleum hydrocarbon-biodegrading activity of this group have been published. The opposite is true for a bacterium of the *Alcanivorax* genus that is known to be involved in the biodegradation of a wide range of hydrocarbons^89^. Among the dominant contaminated soil bacteria that were identified to a species or genus, *Rhodanobacter ginsengisoli* is notable. Representatives of the genus *Rhodanobacter* have been found in bacterial consortia involved in the biodegradation of crude oil^90^. The dominant bacteria also included *Acidobacterium capsulatum*, a chemoorganotrophic microorganism that has not been previously noted among oil-degrading bacteria but is known to be capable of living in a wide range of concentrations of various carbon sources^91^, and *Acidocella,* which can metabolize several aromatic hydrocarbons^92^.

Comparing the presented data with the results obtained by other authors in research on oil-contaminated soils, two conclusions can be noted. First, contamination often leads to the dominance of the same hydrocarbon-degrading microorganisms, regardless of the conditions, extent and history of contamination or soil properties. Second, combinations of dominant taxa and their hierarchical rank can dramatically differ even with small variations of the listed conditions^74^. A decisive role is played by the composition of the microbial community before contamination, since consortia that efficiently utilize hydrocarbons can be built from microorganisms that are present in natural soil, even at a very low abundance. In addition, settlement/resettlement processes can also be very active and play a role in the formation of functional communities. These two assumptions are supported by the NMDS analysis results (Fig. 3A) and the Venn diagram (Fig. 4B) showing that the composition of bacteria detected by high-throughput sequencing changes by more than half as a result of contamination and differs significantly depending on whether remediation was performed or not.

#### Fungi

The richness of representatives of the fungi kingdom in the study area varied greatly. For example, in the samples from the undisturbed site, the number of OTUs in one sample varied from 126 to 314; at the recultivation site, it varied from 96 to 421. The richness of fungi at the SR site was significantly lower: the number of OTUs in one sample ranged from 51 to 191. The lower richness of fungi at the most contaminated site was confirmed by the values of the Chao index (Fig. 5B), which were significantly lower than those at the undisturbed and recultivation sites. The Shannon index demonstrated exactly the same trends as the Chao index; however, the differences in this case were not statistically significant (Fig. 5B).

The NMDS plot (Fig. 3B) shows a clustering of samples from the UF site separately from the R and SR sites. Points of oil-contaminated sites form two distinct zones located close to each other. The R site points are in an intermediate position between the most contaminated SR site and the undisturbed community of the UF site, as in the case of bacterial communities.

The fungal community (Fig. 5A) in the study area was represented predominantly by the Ascomycota, Basidiomycota and Rozellomycota phyla. Almost 80% of the community of the undisturbed site consisted of ascomycetes; basidiomycetes comprised 18.9% of all fungi, and the share of other phyla was insignificant. As in the present study, the dominance of Ascomycota was noted in temperate forests of North America^75,76,93^.

The structure of the fungal community at the oil-contaminated sites was different (Fig. 5C). The OTU number of Ascomycota at the most oil-contaminated SR site was almost two times lower. At the R site, which was less contaminated with oil products, the richness of ascomycetes was slightly higher than that at the UF site. The abundance of this phylum at the SR site was the same as that at the UF site, and the abundance at the R site tended to be lower than that at the undisturbed site. Representatives of Basidiomycota at the UF site accounted for approximately one-fifth of all fungi; at the oil-contaminated sites, their abundance was much lower. A decrease in the richness of these saprotrophic fungi was observed at both contaminated sites, but it was significant only at the SR site.

In addition to the significant abundance of unknown species at the oil-contaminated sites, a significant reorganization of the ratio of the species of fungi of the Ascomycota and Basidiomycota phyla was revealed.

In the composition of *Ascomycota* (Table 4), which dominated at all sites, representatives of the Leotiomycetes (44.3%) and Lecanoromycetes (30.4%) classes were predominant at the undisturbed site (the dominance of the Lecanoromycetes was due to the high representation of one OTU (OTU_1, an unclassified Lecanorales). Among Leotiomycetes, more than 90% were representatives of the family Helotiaceae, saprotrophic fungi involved in the decomposition of plant remnants, which also includes plant parasites and predatory fungi. Both the share of Leotiomycetes and their OTU number were significantly higher at the R site and lower at the SR site. Lecanoromycetes, the dominating OTU_1, had almost negligible abundance at the contaminated SR and R sites; this species was replaced by representatives of the Eurotiomycetes class at the R site and by unclassified Ascomycota at the SR site.

**Table 4:** Abundance of OTUs from different Ascomycota classes registered at the study sites (mean ± SEM). Different letters indicate a significant difference among data within the row.

Class/site	Average abundance, %			Average number of OTUs		
	SR	R	UF	SR	R	UF
Eurotiomycetes	7. 6 ± 2.1^a^	32.5 ± 6.3^b^	13.2 ± 2.2^a^	12.1 ± 1.3	22.3 ± 2.3	17.9 ± 1.7
Leotiomycetes	30.9 ± 5.3	52.3 ± 5.3	44.3 ± 6.1	27.4 ± 2.5^a^	75.9 ± 8.8^b^	65.8 ± 3.9^b^
Sordariomycetes	3.5 ± 1.7^a^	0. 5 ± 0.1^ab^	0.4 ± 0.2^b^	8.0 ± 0.7	9.6 ± 0.9	5.6 ± 0.9
Dothideomycetes	3.0 ± 1.4	1.2 ± 0.3	1.4 ± 0.6	4.9 ± 1.0	11. 7 ± 2.1	9.0 ± 1.4
Lecanoromycetes	0.05 ± 0.02^a^	0.8 ± 0.5^a^	30.4 ± 6.6^b^	0.7 ± 0.2^a^	2.1 ± 0.5^a^	3.9 ± 0.3^b^
Unclassified_Ascomycota	54.9 ± 4.3^a^	12.7 ± 3.9^b^	9.8 ± 2.9^b^	12. 7 ± 0.9^a^	27.5 ± 3.6^b^	17.5 ± 1.7^ab^

The share of another Ascomycota class of fungi, Sordariomycetes, was 8 times higher at the SR site than at the R and UF sites.

Among Basidiomycota, 97% at the undisturbed site were representatives of the class Agaricomycetes (Table 5), which is apparently typical of temperate forests^76^. These species of fungi can be both saprotrophs and parasites, as well as form mycorrhizas. Basidiomycetes of the recultivation site were represented by three classes: Agaricomycetes, Microbotryomycetes and Tremellomycetes. The representatives of Microbotryomycetes were predominant at the self-restoration site. Some representatives of this class of fungi are noted for being able to degrade petroleum hydrocarbons^94,95^.

**Table 5:** Abundance of OTUs from different Basidiomycota classes registered at the study sites (mean ± SEM). Different letters indicate a significant difference among data within the row.

Class/site	Average abundance, %			Average number of OTUs		
	SR	R	UF	SR	R	UF
Agaricomycetes	19.5 ± 4.9^a^	35.0 ± 5.9^a^	97.2 ± 0.9^b^	8.3 ± 1.5^a^	13.6 ± 2.4^a^	29.8 ± 3.6^b^
Microbotryomycetes	49.4 ± 6.2^a^	28.2 ± 5.2^a^	0.03 ± 0.01^b^	6.1 ± 0.7^a^	5.8 ± 0.9^a^	1.0 ± 0.3^b^
Tremellomycetes	11.3 ± 2.8^a^	33.7 ± 5.9^b^	1.2 ± 0.4^c^	3.8 ± 0.4	6.9 ± 1.0	4.1 ± 0.4
Exobasidiomycetes	6.4 ± 3.1	0.03 ± 0.02	0	0.5 ± 0.1	0.3 ± 0.2	0
Cystobasidiomycetes	0.1 ± 0.1	1.6 ± 1.0	0	0.3 ± 0.2	0.8 ± 0.2	0
Unclassified Basidiomycota	12.9 ± 5.3	0.8 ± 0.3	1.6 ± 0.5	1.4 ± 0.2^a^	1.1 ± 0.2^a^	4.5 ± 0.5^b^

Unfortunately, there is very little research on changes in fungal communities in oil-contaminated soils. For instance, among identified strains isolated from contaminated soils of Kazakhstan, the predominance of Ascomycota (78%), capable of biodegradation of oil hydrocarbons, was identified; representatives of Basidiomycota comprised 22%^85^.

A general view of the changes in the composition of the dominant fungal OTUs at the study sites yields similar conclusions as in the case of the dominant OTUs in the bacterial community. The composition of the most abundant species in the contaminated soils completely changed, despite the lack of dramatic differences in the integral indicators of biodiversity (Fig. 6A).

The predominance of an uncultured representative of Ascomycota in the soil of the most contaminated SR site was noted (on average, 21.3% of all ITS reads in samples of the SR site). This species, as well as the next most abundant representatives of Ascomycota fungi and one representative of an unidentified group, can be important in the processes of utilization of oil hydrocarbons or may have the greatest resistance to their presence in the soil. In any case, these taxa require further study.

Second in abundance in soil of the highly contaminated SR site was *Amorphotheca resinae*, another Ascomycota. This species is capable of growing in containers with aviation fuel and is a well-known hydrocarbon destructor^96^. It is noteworthy that the abundance of this species at the R and UF sites was similar and extremely low (0.00148% and 0.00143%, respectively). Thus, only a very high level of pollution could have led to the dominance of this species.

Oil contamination led to deep reorganization of the community. The dominant OTU_1 at the UF site was almost completely absent at the oil-contaminated sites SR and R. The richness of Ascomycota at the most oil-contaminated SR site was two times lower than that at the UF site. At the same time, the abundance of this phylum at the SR site was higher than that at the undisturbed site, especially for unclassified Ascomycota and the class Sordariomycetes. Conversely, the number of OTUs of ascomycetes at the less oil-contaminated R site was slightly higher and the abundance was generally significantly lower. Agaricomycetes, which comprised 97% of Basidiomycota at the undisturbed site, were dramatically reduced at the oil-contaminated sites, and the diversity of this phylum of fungi at the SR and R sites was 2- and threefold lower, respectively. Species of the Microbotryomycetes class were dominant at the SR site.

## Conclusion

This study provides the first description of the composition of soil bacterial and fungal communities using marker fragments of the 16S rRNA gene and ITS region at European Subarctic sites with an increased content of oil products and variation in recultivation and self-restoration. The microbial community at oil-contaminated sites was found to be reorganized at the phylum level: the shares of Proteobacteria and Actinobacteria were higher whereas the share of Acidobacteria was lower than that at the background site. In fungal communities, the richness of the Ascomycota class was lower than that at the background site. Moreover, a decrease in the OTU number and a complete change in the composition of the dominant OTUs among both bacteria and fungi occurred at the contaminated sites.

Among the bacterial genera studied, there was a manifold increase in the abundance of known degraders of hydrocarbons at the oil-contaminated sites compared with the background site: *Alcanivorax* (SR: 5.35%; R: 0.2%; UF: 0.005%), *Rhodanobacter ginsengisoli* (SR: 2.9%; R: 2.2%; UF: 0.5%), *Acidobacterium capsulatum* (SR: 2.5%; R: 0.11%; UF: 0.01%), and *Acidocella* (SR: 1.2%; R: 0.2%; UF: 0.07%). Among the fungi, an increase in the abundance of *Amorphotheca resinae* (SR: 11.4%; R: 0.00148%; UF: 0.00143%) was observed. However, the dominant positions in soils with the highest level of contamination were occupied by an uncultivated representative of the Chromatiales order (SR: 6.45%; R: 0.12%; UF: not detected) among bacteria and by representatives of the Ascomycota phylum (SR: 21.28%; R: 0.3%; UF: 0.0004%) among fungi, which were almost absent in the background soils. Possible oil-destructive properties or high resistance to oil contamination make these organisms important for further study.

Despite the very large differences between the sites studied, mechanical recultivation was generally found to be relatively efficient in terms of reducing the amount of oil products and preventing a decrease in the taxonomic richness of bacteria and fungi.

At the recultivation and self-restoration sites, the communities of indicator taxonomic groups of soil microfauna (oribatid mites and springtails) demonstrated an extreme degree of their degradation, which was manifested by the low total abundance, small number of species, and absence of euedaphic forms of collembolans. Among oribatid mites, the eurytopic species *Oppiella nova* was the most abundant; among springtails, *Proisotoma minima* was the most dominant species due to its tolerance of a high level of soil contamination.

The data obtained contribute to the extremely limited knowledge about the microbiota and microfauna of subarctic soils and their response to heavy oil pollution and the effectiveness of mechanical recultivation. The accumulation and systematization of such data are necessary to develop approaches for monitoring and restoring oil-polluted ecosystems. It will be useful to analyse the effectiveness of other existing methods of recultivation to select the optimal method for a given climate and soil type. The uncultivated dominant species found in the contaminated samples are of interest for further study as potential new oil degraders.

## Supplementary Information

Supplementary Information 1.

Supplementary Information 2.

Supplementary Information 3.

## Data Availability

The metataxonomics data sets generated during the current study are available in the NCBI repository (registration number PRJNA661725). https://www.ncbi.nlm.nih.gov/bioproject/661725. All other data generated during this study are included in this published article and its Supplementary Information files.
