# Supplementary material for: Soil microbiota and microarthropod communities in oil contaminated sites in the European Subarctic
Source: Sci Rep. 2021 Oct 4;11:19620. doi: 10.1038/s41598-021-98680-8 (PMC8490368; doi:10.1038/s41598-021-98680-8)
Supplement: Supplementary file 1 — Supplementary Information 1. [file 41598_2021_98680_MOESM1_ESM.docx]

**Table S1.** Species list and abundance (individuals m^-2^ ± SEM) of oribatid mites found in soil of the contaminated (SR, R) and uncontaminated (UF)

| Family/Species | Site SR | Site R | Site UF |
| --- | --- | --- | --- |
| Palaeacaridae / *Palaeacarus hystricinus* Trägårdh, 1932 |  |  | 267 ± 186 |
| Hypochthoniidae / *Hypochthonius rufulus* Koch, 1835 |  |  | 167 ± 167 |
| Brachychthoniidae / *Liochthonius (L.) sellnicki* (Thor, 1930) |  |  | 767 ± 360 |
| Phthiracaridae / *Atropacarus (A.) striculus* (Koch, 1835) |  |  | 167 ± 115 |
| Euphthiracaridae / *Euphthiracarus (E.) cribrarius* s. str. (Berlese, 1904) |  |  | 33 ± 33 |
| Nothridae / *Nothrus pratensis* Sellnick, 1928 |  |  | 67 ± 67 |
| Crotoniidae / *Heminothrus (H.) longisetosus* (Willmann, 1925) |  |  | 800 ± 171 |
| “” / *Camisia (C.) biurus* (Koch, 1839) |  |  | 167 ± 92 |
| Malaconothridae / *Malaconothrus (M.) monodactylus* (Michael, 1888) |  |  | 333±333 |
| Nanhermanniidae / *Nanhermannia (N.) sellnicki* Forsslund, 1958 |  |  | 4933±1223 |
| Damaeidae / *Damaeus (E.) bituberculatus* (Kulczynski, 1902) |  | 33±33 | 733±315 |
| Eremaeidae / *Eueremaeus oblongus silvestris* (Forsslund, 1956) |  |  | 2967±1378 |
| Ceratoppiidae / *Ceratoppia bipilis* s. str. (Hermann, 1804) |  |  | 100±100 |
| Tectocepheidae / *Tectocepheus velatus* s. str. (Michael, 1880) |  | 33±33 | 26333±6540 |
| Oppiidae / *Moritzoppia unicarinata* s. str. (Paoli, 1908) |  |  | 3200±786 |
| “” / *Oppiella (M.) neerlandica* (Oudemans, 1900) |  |  | 10567±4122 |
| “” / *Oppiella (O.) nova* s. str. (Oudemans, 1902) | 833±476 | 767±267 | 11467±2538 |
| Suctobelbidae / *Suctobelbella (*S.*) acutidens* s. str. (Forsslund, 1941) |  |  | 3967±770 |
| Quadroppiidae / *Quadroppia (Q.) quadricarinata* (Michael, 1885) |  |  | 2800±507 |
| Carabodidae / *Carabodes (C.) labyrinthicus* (Michael, 1879) |  |  | 133±103 |
| Scheloribatidae / *Scheloribates (S.) laevigatus* s. str. (Koch, 1835) | 167±59 | 33±33 | 3300±995 |
| Oribatulidae / *Oribatula (Zygoribatula) exilis* s. str. (Nicolet, 1855) |  | 33±33 |  |
| “” / *Oribatula (O.) tibialis* (Nicolet, 1855) | 33±33 |  | 1400±529 |
| Ceratozetidae / *Ceratozetes (C.) gracilis* s. str. (Michael, 1884) |  |  | 6033±1429 |
| “” / *Melanozetes mollicomus* (Koch, 1839) |  |  | 600±244 |
| “” / *Edwardzetes (E.) edwardsi* (Nicolet, 1855) |  |  | 567±258 |
| Humerobatidae / *Diapterobates variabilis* s. str. Hammer, 1955 |  |  | 433±115 |
| Chamobatidae / *Chamobates (C.) pusillus* (Berlese, 1895) |  |  | 2467±642 |
| Phenopelopidae / *Eupelops plicatus* (Koch, 1835) |  |  | 767±367 |
| Galumnidae / *Pergalumna (P.) nervosa* s. str. (Berlese, 1914) |  |  | 167±104 |
| Species number | 3 | 5 | 29 |
| Total abundance | 1033±486^a^ | 900±296^a^ | 85700±13145^b^ |
| Species richness | 0.9±0.2^a^ | 0.8±0.2^a^ | 15.3±0.7^b^ |
| Shannon index | 0.1±0.1 | 0.09±0.09 | 1.93±0.08 |
| Life forms |  |  |  |
| Epiedaphic | **̶** | 33±33^a^ | 20067±2619^b^ |
| Hemiedaphic | **̶** | ̶ | 1067±227 |
| Euedaphic | 833±476^a^ | 767±267^a^ | 32000±7304^b^ |
| Eurybiontic | 200±60^a^ | 100±52^a^ | 31033±6691^b^ |
| Non-specialized | **̶** | **̶** | 1200±415 |
| Hydrobiontic | **̶** | **̶** | 333±333 |

^a, b, c^ - Hereinafter, in the tables, uppercase letters denote statistical differences between the corresponding indicators in different experimental sites (Mann-Whitney U-test). “” – denote same family as above.

**Table S2.** Species list and abundance (individuals m^-2^ ± SEM) of springtails found in the soil of the contaminated (SR, R) and uncontaminated (UF)

| Family / Species | Site SR | Site R | Site UF |
| --- | --- | --- | --- |
| Tullbergiidae / *Mesaphorura macrochaeta* Rusek, 1976 |  |  | 800±295 |
| Onychiuridae / *Oligaphorura*  *absoloni* Börner, 1901 |  |  | 467±183 |
| “” / *Protaphorura boedvarssoni* Pomorski, 1993 |  |  | 867±273 |
| “” / *Protaphorura subarctica* (Martynova, 1976) |  |  | 2700±424 |
| “” / *Supraphorura furcifera* Börner, 1901 |  |  | 67±45 |
| Hypogastruridae / *Ceratophysella denticulata* (Bagnall, 1941) | 167±115 |  |  |
| “” / *Willemia anophthalma* Börner, 1901 |  |  | 1933±464 |
| Neanuridae / *Anurida ellipsoides* Stach, 1949 |  |  | 167±134 |
| “” / *Friesea truncata* Cassagnau, 1958 |  |  | 1067±536 |
| “” / *Micranurida pygmaea* Börner, 1901 |  |  | 1233±731 |
| Odontellidae */ Xenyllodes armatus* Axelson, 1903 |  | 364±143 |  |
| Isotomidae / *Desoria hiemalis* (Schött, 1893) |  | 436±197 |  |
| “” / *D. neglecta* (Schäffer, 1900) | 33±33 | 36±33 |  |
| “” / *Folsomia manolachei* (Bagnall, 1939) |  |  | 45667±10014 |
| “” / *F. quadrioculata* (Tullberg, 1871) | 233±125 |  |  |
| “” / *Isotoma viridis* Bourlet, 1839 | 33±33 |  |  |
| “” / *Isotomiella minor* (Schäffer, 1896) |  |  | 3400±650 |
| “” / *Parisotoma notabilis* (Schäffer, 1896) | 67±67 | 109±100 | 100±72 |
| “” / *Proisotoma minima* (Absolon, 1901) | 13333±2344 | 800±278 |  |
| “” / *Pseudanurophorus binoculatus* (Kseneman, 1934) |  |  | 267±102 |
| “” / *Pseudisotoma sensibilis* (Tullberg, 1876) |  |  | 100±72 |
| Neelidae / *Megalothorax minimus* Willem, 1900 |  |  | 33±33 |
| Sminthurididae / *Sphaeridia pumilis* (Krausbauer, 1898) |  |  | 67±45 |
| Bourletiellidae / *Heterosminthurus claviger* Gisin, 1958 |  | 36±33 |  |
| Total abundance | 13867±2341^a^ | 1800±551^b^ | 58933±10406^c^ |
| Species richness | 1.7±0.3^a^ | 2.1±0.3^a^ | 7.8±0.5^b^ |
| Shannon index | 0.17±0.07 | 0.57±0.13 | 1.01±0.12 |
| Life forms |  |  |  |
| Epiedaphic | 233±134^a^ | 833±293^ab^ | 1233±551^b^ |
| Hemiedaphic | 13633±2398^a^ | 967±293^b^ | 47433±10339^c^ |
| Euedaphic |  |  | 10267±1340 |
| Trophic groups |  |  |  |
| EPMC | 67±45 | 467±219 | 167±77 |
| EAMC | 167±115^a^ | 367±143^ab^ | 1233±522^b^ |
| HMC | 13633±2398^a^ | 967±293^b^ | 49433±10154^c^ |
| EMC |  |  | 4100±635 |
